# Foliar application of iron-lysine to boost growth attributes, photosynthetic pigments and biochemical defense system in canola (*Brassica napus* L.) under cadmium stress

**DOI:** 10.1186/s12870-023-04672-3

**Published:** 2023-12-16

**Authors:** Mohammad K. Okla, Muhammad Hamzah Saleem, Ibrahim A. Saleh, Naser Zomot, Shagufta Perveen, Abida Parveen, Fozia Abasi, Habib Ali, Baber Ali, Yasmeen A. Alwasel, Mostafa A. Abdel-Maksoud, Mükerrem Atalay Oral, Sadia Javed, Sezai Ercisli, Muhammad Hassan Sarfraz, Mahdy H. Hamed

**Affiliations:** 1 Department of Botany and Microbiology, College of Science, King Saud University, Riyadh, 11451 Saudi Arabia; 2 College of Plant Science and Technology, Huazhong Agricultural University, Wuhan, 430070 China; 3 Faculty of Science, Zarqa University, Zarqa, 13110 Jordan; 4 Department of Botany, Government College University, Faisalabad, 38000 Pakistan; 5 Department of Agronomy, PMAS-Arid Agriculture University, Rawalpindi, 46300 Pakistan; 6 Department of Plant Sciences, Quaid-i-Azam University, Islamabad, 45320 Pakistan; 7 Elmalı Vocational School of Higher Education, Akdeniz University, Antalya, 07058 Türkiye; 8 Department of Horticulture, Agricultural Faculty, Ataturk University, Erzurum, 25240 Türkiye; 9 HGF Agro, Ata Teknokent, Erzurum, TR-25240 Türkiye; 10 Nuffield Department of Orthopaedics, Rheumatology and Musculoskeletal Sciences, Botnar Institute of Musculoskeletal Sciences, University of Oxford, Oxford, OX3 7LD UK; 11 Department of Soils and Water, Faculty of Agriculture, New Valley University, Kharga, 72511 Egypt; 12 Department of Botany, PMAS-Arid Agriculture University, Rawalpindi, 46300 Pakistan

**Keywords:** Micro-chelation application, Heavy metal contamination, Chlorophyll pigments, Proline

## Abstract

In the current industrial scenario, cadmium (Cd) as a metal is of great importance but poses a major threat to the ecosystem. However, the role of micronutrient − amino chelates such as iron − lysine (Fe − lys) in reducing Cr toxicity in crop plants was recently introduced. In the current experiment, the exogenous applications of Fe − lys i.e., 0 and10 mg L − 1, were examined, using an in vivo approach that involved plant growth and biomass, photosynthetic pigments, oxidative stress indicators and antioxidant response, sugar and osmolytes under the soil contaminated with varying levels of Cd i.e., 0, 50 and 100 µM using two different varieties of canola i.e., Sarbaz and Pea − 09. Results revealed that the increasing levels of Cd in the soil decreased plant growth and growth-related attributes and photosynthetic apparatus and also the soluble protein and soluble sugar. In contrast, the addition of different levels of Cd in the soil significantly increased the contents of malondialdehyde (MDA) and hydrogen peroxide (H2O2), which induced oxidative damage in both varieties of canola i.e., Sarbaz and Pea − 09. However, canola plants increased the activities of superoxide dismutase (SOD), peroxidase (POD), catalase (CAT), and non-enzymatic compounds such as phenolic, flavonoid, proline, and anthocyanin, which scavenge the over-production of reactive oxygen species (ROS). Cd toxicity can be overcome by the supplementation of Fe − lys, which significantly increased plant growth and biomass, improved photosynthetic machinery and sugar contents, and increased the activities of different antioxidative enzymes, even in the plants grown under different levels of Cd in the soil. Research findings, therefore, suggested that the Fe − lys application can ameliorate Cd toxicity in canola and result in improved plant growth and composition under metal stress.

## Introduction

The widespread use of farming practices and industrialization has led to an abrupt increase in the level of potentially toxic elements that adversely affect crop production worldwide [1, 2]. The major heavy metals, such as cadmium (Cd), chromium (Cr), zinc (Zn), aluminum (Al), copper (Cu), mercury (Hg), and lead (Pb) are known to exhibit phytotoxicity, affecting plant growth and development [3, 4]. Cadmium, due to its high solubility, mobility and efficient soil-plant transfer, has emerged as a significant threat to all living organisms [5–7]. Cadmium in the soil is taken up by plant roots and eventually reaches humans through the food chain, representing health risks to the immune, nervous, and reproductive systems [8–10]. Chlorophyll plays a crucial role in photosynthesis, and any impairment in chlorophyll biosynthesis in response to Cd stress obstructs the photosynthetic machinery in the major crops [11–13]. Moreover, higher Cd retention in plant cells/tissues triggers the production of reactive oxygen species (ROS), hydroxyl groups (OH) and superoxide radicles (O^•−^) which either directly or indirectly affects the in planta metabolic pathways [14–16]. Over-production of ROS is toxic and plants needs to scavenge those immediately through antioxidative defense system [17, 18]. Clearly, a viable and cost-effective method to remove Cd from the environment is needed.

To date, many scientific approaches have been employed to remediate the metal stress in plants, particularly by using exogenous applied amino acids. Amino acids play a very critical role in metal compartmentation, transport and tolerance in plants. Zinc (Zn) and iron (Fe) chelated fertilizers complexed with lysine (lys) as amino acid have been reported to improve the growth and yield of crops [19–21]. Fruitful application of micronutrients expressively mitigates the toxic effects of metal stress in plants [22, 23]. Fe is an important micronutrient as they play a very critical role in various metabolic processes in plants including photosynthesis, DNA synthesis and respiration [24, 25]. Fe chelated with the amino acid lys effectively mitigates the metal stress in *Brassica napus* and *Spinacia oleracea* and improved the growth of plant through Fe fortification [23, 26]. However very little is known about the role of Fe − lys regarding the mitigation of Cd stress in canola. Canola (*Brassica napus* L.), a member of the *Brassicaceae* family with 325 genera and 3740 species, holds a prominent position as a global oilseed crop, primarily cultivated in Canada, Europe, and Australia, with an annual output of 72 million tonnes [27]. Canola showed a great resistance to heavy metal stress, however wide concentration of heavy metals prompted severe stress in canola [28–30]. It is well recognized that Cd stress significantly affected the molecular, ultra-structural and physiochemical profiling of canola plants [31, 32].

Cultivation of cereal crops in metal polluted sites marks the unwanted buildup of Cd in different vegetables and crops like spinach [24, 33], rice [2, 34] and wheat [35, 36] but very few literatures are available on canola grown in metal contaminated site. The results from the present findings will add to our knowledge about (i) the role of Fe–lys on plant growth, fresh weights, dry weights, chlorophyll contents, gaseous exchange attributes, oxidative stress and antioxidant response and (ii) sugar and osmolytes response when cultivated in the sites which are rich with Cd concentration. According to best of our knowledge, this study is among the few studies which focus on the metal tolerance and accumulation among oil seed crops in order to investigate their suitability for metal–contaminated sites. Findings from the present study will add to our understanding the mechanism of Cd tolerance and accumulation in canola with the foliar application of Fe–lys.

## Materials and methods

### Experimental design

Soil was collected from agricultural field at the depth of 0–20 cm using a stainless–steel blade. The soil was thoroughly sieved to 2 mm in order to completely remove the unwanted materials such as previous crop residues and debris. Then, the soil was analyzed for physicochemical properties. Concentration of organic matter in soil was determined with the method recommended by Walkley and Black [37]. Hydrometer was used for the efficacious analysis of soil texture [38]. Sodium adsorption ratio (SAR), electrical conductivity (EC), and soluble ions were also measured [39]. Ammonium bicarbonate diethylenetriamine pentaacetic acid (AB-DTPA) extractable in soil was estimated with the method described by Soltanpour [40]. The complete detail of physicochemical characteristics of the soil used for the pot experiment.

### Pot experiment

The present experiment was carried out in the pots and each pot was filled with 5 kg of soil. Seeds of two different varieties of canola i.e., Sarbaz and Pea − 09 were used in this study and the seeds were surface sanitized with H_2_O_2_ solution and washed 5 times with distilled water before sowing in pots. Two weeks after germination, four morphologically uniform seedlings were selected for further study. Before starting the pot experiment, the soil was artificially spiked with varying levels of Cd i.e., 0, 50 and 100 µM by using CdCl_2_ salt. All pots have undergone two cycles of water saturation and air drying. Different levels of foliar treatments of Fe–lys (0, 10 mg L^− 1^) were used in this study, with the help of hand sprayer. The same levels of Fe–lys was used in a previous study by Zaheer et al. [41]. While the Cd concentration used in this study was less than our previous conduced experiment [15] in maize. A total of 4–L volume of Fe–lys was applied through four foliar applications per treatment, and plants under control treatment were sprayed carefully with distilled water. Soil used during the pot experiment was properly covered to avoid the needless contamination. All Fe–lys and wastewater treatments were applied in split doses to avoid any sudden toxicity to plants, and a completely randomized design of the experiment was followed with four replicates of each treatment. The appropriate amount of fertilizers was applied as recommended by Bashir et al. [22]. Experimental pots were regularly rotated and weeds were removed manually.

### Growth attributes

Experimental data were collected from the propagated shoot and root lengths, as well as the weight of fresh and dry leaves of the two cultivars, which were recorded. Root length and shoot length was measured using a measuring scale from the tips of the shoots to the bottom of the root tips. After that, fresh biomass (roots and shoots) was also measured using a weighting digital balance by selecting three randomly plants per treatment. The plant samples were oven-dehydrated at 65 °C for 72 h for Cd and ions concentration determination and the total plant dry weight was also measured. Before being oven-dried, roots were immersed in 20 mM Na_2_EDTA for 15–20 min to remove Cd that had adhered to the surface of roots. Subsequently, roots were washed thrice with distilled water and finally once with deionized water then dried for further analysis.

### Photosynthetic pigments

Photosynthetic Pigments Leaves were collected for the determination of chlorophyll and carotenoid contents. For chlorophyll, 0.1 g of fresh leaf sample was extracted using 8 mL of 95% acetone for 24 h at 4 °C under dark conditions. The absorbance was measured by a spectrophotometer (UV-2550; Shimadzu, Kyoto, Japan) at 646.6, 663.6, and 450 nm. Chlorophyll content was calculated using the standard method of Arnon [42].

### Oxidative stress indicators

The degree of lipid peroxidation was evaluated as malondialdehyde (MDA) contents. Briefly, 0.1 g of frozen leaves were ground at 4 °C in a mortar with 25 mL of 50 mM phosphate buffer solution (pH 7.8) containing 1% polyethene pyrrole. The homogenate was centrifuged at 10,000× g at 4 ◦C for 15 min. The mixtures were heated at 100 °C for 15–30 min and then quickly cooled in an ice bath. The absorbance of the supernatant was recorded by using a spectrophotometer (xMark™ Microplate Absorbance Spectrophotometer; Bio-Rad, Hercules, CA, USA) at wavelengths of 532, 600, and 450 nm. Lipid peroxidation was expressed as 1 mol g^− 1^ by using the formula: 6.45 (A532 − A600) − 0.56 A450. Lipid peroxidation was measured using a method previously published by Heath and Packer [43].

To estimate H_2_O_2_ content of plant tissues, 3 mL of sample extract was mixed with 1 mL of 0.1% titanium sulfate in 20% (v/v) H_2_SO_4_ and centrifuged at 6000× g for 15 min. The yellow color intensity was evaluated at 410 nm. The H_2_O_2_ level was computed by the extinction coefficient of 0.28 mmol^− 1^ cm^− 1^. The contents of H_2_O_2_ were measured by the method presented by Jana and Choudhuri [44].

### Activities of antioxidant enzymes

To evaluate enzyme activities, fresh leaves (0.5 g) were homogenized in liquid nitrogen and 5 mL of 50 mmol sodium phosphate buffer (pH 7.0), including 0.5 mmol EDTA and 0.15 mol NaCl. The homogenate was centrifuged at 12,000× g for 10 min at 4 °C, and the supernatant was used for measurement of superoxidase dismutase (SOD) and peroxidase (POD) activities.

SOD activity was assayed in a 3 mL reaction mixture containing 50 mM sodium phosphate buffer (pH 7), 56 mM nitro blue tetrazolium, 1.17 mM riboflavin, 10 mM methionine, and 100 µL enzyme extract. Finally, the sample was measured using a spectrophotometer (xMark™ Microplate Absorbance Spectrophotometer; Bio-Rad). Enzyme activity was measured by using a method employed by Chen and Pan [45] and expressed as U g^− 1^ FW. POD activity in the leaves was estimated by using the method of Sakharov and Ardila [46] employed by using guaiacol as the substrate. A reaction mixture (3 mL) containing 0.05 mL of enzyme extract, 2.75 mL of 50 mM phosphate buffer (pH 7.0), 0.1 mL of 1% H_2_O_2_, and 0.1 mL of 4% guaiacol solution was prepared. Increases in the absorbance at 470 nm due to guaiacol oxidation was recorded for 2 min. One unit of enzyme activity was defined as the amount of the enzyme.

Catalase (CAT) activity was analyzed according to Aebi [47]. The assay mixture (3.0 mL) was comprised of 100 µL enzyme extract, 100 µL H_2_O_2_ (300 mM), and 2.8 mL 50 mM phosphate buffer with 2 mM ETDA (pH 7.0). The CAT activity was measured by the decline in absorbance at 240 nm as a result of H_2_O_2_ loss (ε = 39.4 mM^− 1^ cm^− 1^).

### Non-enzymatic antioxidants, sugars, and proline contents

Plant ethanol extracts were prepared for the determination of nonenzymatic antioxidants and some key osmolytes. For this purpose, 50 mg of dry plant material was homogenized with 10 mL ethanol (80%) and filtered through Whatman No. 41 filter paper. The residue was reextracted with ethanol, and the 2 extracts were pooled together to a final volume of 20 mL. The determination of flavonoids [48], phenolics [49], anthocyanin [50], and total sugars [51] was performed from the extracts.

Fresh leaf material (0.1 g) was mixed thoroughly in 5 mL of aqueous sulfosalicylic acid (3%). The mixture was centrifuged at 10,000 × g for 15 min, and an aliquot (1 mL) was poured into a test tube containing 1 mL of acidic ninhydrin and 1 mL of glacial acetic acid. The reaction mixture was first heated at 100 °C for 10 min and then cooled in an ice bath. The reaction mixture was extracted with 4 mL of toluene, and the test tubes were vortexed for 20 s and cooled. Thereafter, the light absorbance at 520 nm was measured by using a UV–VIS spectrophotometer (Hitachi U-2910, Tokyo, Japan). The free proline content was determined on the basis of the standard curve at 520 nm absorbance and expressed as µmol (g FW) ^−1^ [52].

### Statistical analysis

Statistical analysis of data was performed with analysis of variance (ANOVA) by using a statistical program Co-Stat version 6.2, Cohorts Software, 2003, Monterey, CA, USA. All the data obtained were tested by two-way analysis of variance (two-way ANOVA). Thus, the differences between treatments were determined by using ANOVA, and the least significant difference test (*P* < 0.05) was used for multiple comparisons between treatment means. Logarithmic or inverse transformations were performed for data normalization, where necessary, prior to analysis. Pearson’s correlation analysis was performed to quantify relationships between various analyzed variables.

## Results

Effect of foliar application of Fe − lys on plant growth and biomass in both varieties of canola grown under the toxic concentration of Cd in the soil.

**Fig. 1:**
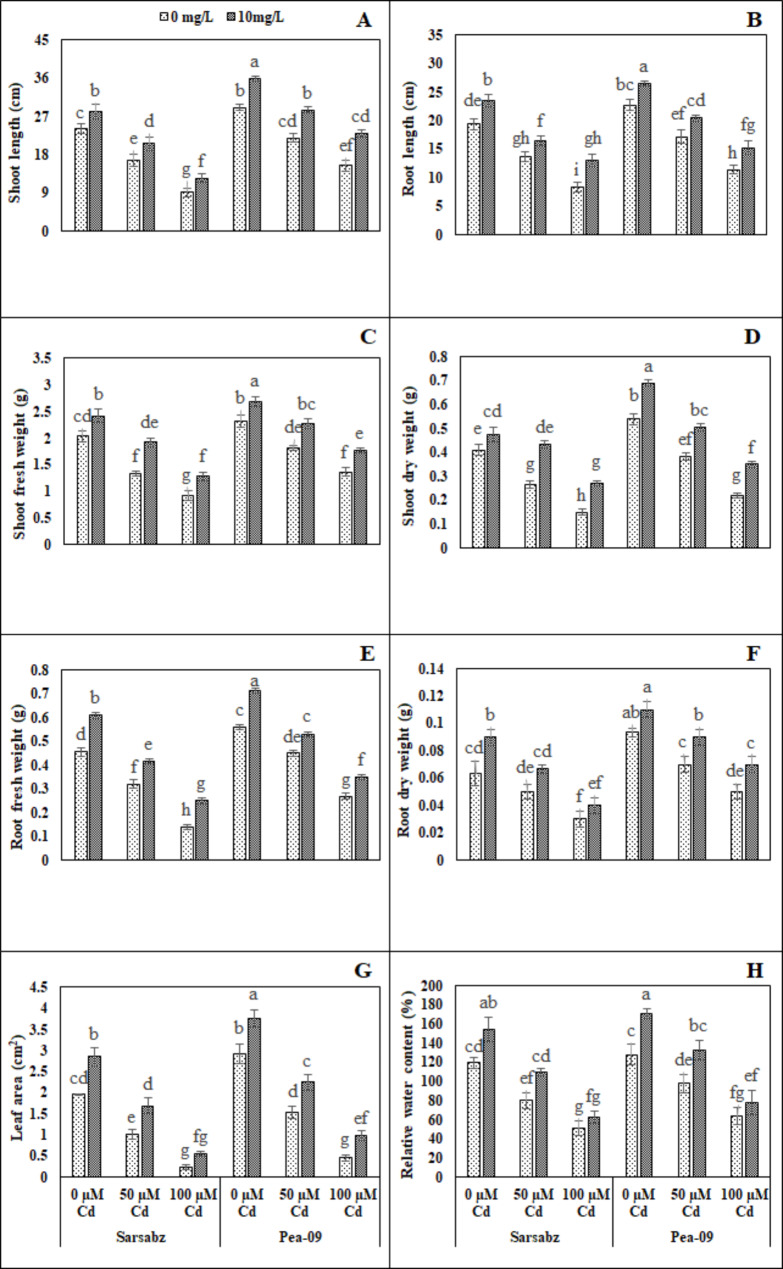
Effect of different levels of Fe − lys (0 and 10 mg L^− 1^) on root length (**A**), shoot length (**B**), root fresh weight (**C**), root dry weight (**D**), shoot fresh weight (**E**), shoot dry weight (**F**), leaf area (**G**), and relative water content (H) under the toxic concentration of Cd (0, 50 and 100 µM) in the soil in both cultivars of canola (Sarbaz and Pea − 09). All the data represented are the average of four replications (n = 4). Error bars represent the standard deviation (SD) of dour replicates. Different lowercase letters on the error bars indicate a significant difference between the treatments

In the present study, various growth and biomass parameters were also measured in both varieties of canola (Sarbaz and Pea − 09) grown under the application of Fe − lys (10 mg L^− 1^) with or without the toxic concentrations of Cd i.e., 0, 50 and 100 µM in the soil. The data regarding root and shoot length, fresh and dry biomass in both varieties of canola is presented in Fig. 1; Table 1. It was noticed that the increasing levels of Cd in the soil significantly (*P* < 0.05) decreased root length, shoot length, root fresh weight, root dry weight, shoot fresh weight, shoot dry weight, leaf area and relative water content compared to the plants which were grown in control. Results showed that the root length, shoot length, root fresh weight, root dry weight, shoot fresh weight, shoot dry weight, leaf area and relative water contents were decreased by 48.78, 50.54, 37.62, 40.96, 39.54, 53.76, 31.63 and 38.07% respectively in Sarbaz while decreased by 38.64, 50.38, 43.65, 34.36, 39.20, 53.08, 42.30 and 40.29% respectively in Pea − 09 compared to the plants which were grown in 0 µM in the soil. Although, plant growth and biomass of canola varieties under the toxic concentration of the Cd in the soil could be improved by the foliar application of Fe − lys (Fig. 1; Table 1). The application of Fe − lys increased the root length, shoot length, root fresh weight, root dry weight, shoot fresh weight, shoot dry weight, leaf area and relative water contents by 21.63, 17.83, 17.06, 9.09%, 13.818, 17.66, 16.14, and 10.15 respectively in Sarbaz, and also increased by 1386.64, 50.38, 39.20, 53.08, 42.3, 49.36, 55.06 and 40.29%, respectively, in the Pea − 09 plants grown under the application of Fe − lys, compared with plants grown in soil without the application of Fe − lys.

**Table 1:** Mean square values from ANOVA of data for Fe-lysine foliar spray modulates growth attributes, photosynthetic pigments, oxidative stress, enzymatic and non-enzymatic antioxidant activities in two *B. napus* cultivars grown under chromium stressed soil

Source	df	SL	Root Length	SFW	SDW	RFW	RDW
**Cultivars (CV)**	1	150.90 ***	98.67 ***	0.03 ***	0.005***	2.60 ***	2.96 **
**Stress (S)**	2	123.63 ***	102.00 ***	0.04 ***	0.004 ***	4.81 ***	1.04 ***
**Treatment (T)**	1	54.51 ***	61.88 ***	0.02 **	0.03 ***	1.50 ***	3.93 **
**CV × S**	2	12.71**	2.42 ns	0.00 ns	0.00 ns	3.40 ns	1.50 ns
**CV × T**	1	2.72 ns	1.00 ns	0.00 ns	0.00 ns	2.05 ns	2.50 ns
**S ×T**	2	2.04 ns	0.95 ns	0.00 ns	0.00 ns	8.33 ns	1.27 ns
**CV × S ×T**	2	0.27 ns	0.15ns	0.00 ns	0.00 ns	6.60 ns	2.43 ns
**Error**	24	1.96	1.67	0.00	0.00	1.21	4.11
**Source**	**df**	**Leaf Area**	**Chl a**	**Chl b**	**T. Chl**	**Carotenoid**	**RWC**
**Cultivars (CV)**	1	2.46 **	2.13 ***	0.02 ***	0.10 ***	4.88 ***	1341.31 ***
**Stress (S)**	2	2.14 ***	1.37 ***	0.009***	0.30 ***	4.50 ***	1525.94 ***
**Treatment (T)**	1	2.12 **	1.17 ***	0.005***	0.10 ***	2.80 **	2124.11 ***
**CV × S**	2	0.03 ns	0.06 ns	0.001***	0.01 ns	2.55 ns	3.40 ns
**CV × T**	1	0.04 ns	0.00 ns	0.00 ns	0.00 ns	3.26 ns	77.81 ns
**S ×T**	2	0.04 ns	0.00 ns	0.00 ns	0.00 ns	1.23 ns	44.01 ns
**CV × S ×T**	2	0.02 ns	0.00 ns	0.00 ns	0.00 ns	3.40 ns	12.53 ns
**Error**	24	0.20	0.02	0.00	0.00	2.33	69.14
**Source**	**df**	**MDA**	**H**_**2**_ **O**_**2**_	**SOD**	**POD**	**CAT**	**Anthocyanin**
**Cultivars (CV)**	1	60.68 *	34.94 *	5355.97 ***	518.52 ***	11664.5 ***	2.62 ***
**Stress (S)**	2	350.86 ***	192.13 ***	2259.73 ***	449.55 ***	8487.0 ***	5.90 ***
**Treatment (T)**	1	53.22 ns	85.52 ***	2458.22 ***	308.24 ***	6618.4 ***	3.05 ***
**CV × S**	2	0.31 ns	1.18 ns	81.28 ns	4.42 ns	306.0 ns	0.05 ns
**CV × T**	1	0.03 ns	0.74 ns	92.81 ns	0.29 ns	71.0 ns	0.07 ns
**S X T**	2	0.95 ns	4.37 ns	30.0 ns	16.94 ns	83.3 ns	0.12 ns
**CV × S ×T**	2	1.18 ns	0.09 ns	21.70 ns	0.93 ns	161.2 ns	0.07 ns
**Error**	24	14.38 ns	6.67	80.00	12.52	278.4	0.20
**Source**	**df**	**Phenolics**	**Flavonoids**	**Proline**	**TSS**	**TSP**	
**Cultivars (CV)**	1	1308.80 ***	1869.69 ***	301.71 ***	152,561 ***	20.57 ***	
**Stress (S)**	2	2305.42 ***	3267.38 ***	535.90***	225,160 ***	8.60 ***	
**Treatment (T)**	1	1699.43 ***	3408.20 ***	229.92 **	163,458 ***	6.72 ***	
**CV × S**	2	46.43 ns	41.36 ns	61.90 ns	1275 ns	0.30 ns	
**CV ×T**	1	22.78 ns	21.90 ns	15.34 ns	5230 ns	0.06 ns	
**S ×T**	2	1.81 ns	177.78 ns	6.60 ns	2835 ns	0.07 ns	
**CV ×S × T**	2	16.62 ns	3.01 ns	6.44 ns	2242 ns	0.11 ns	
**Error**	24	49.21	69.34	22.06	6323	0.20	

*, **, ***= significant at 0.05, 0.01, and 0.001 levels respectively. ns = non-significant

### Effect of foliar application of Fe − lys on photosynthetic pigments in both varieties of canola grown under the toxic concentration of cd in the soil

**Fig. 2:**
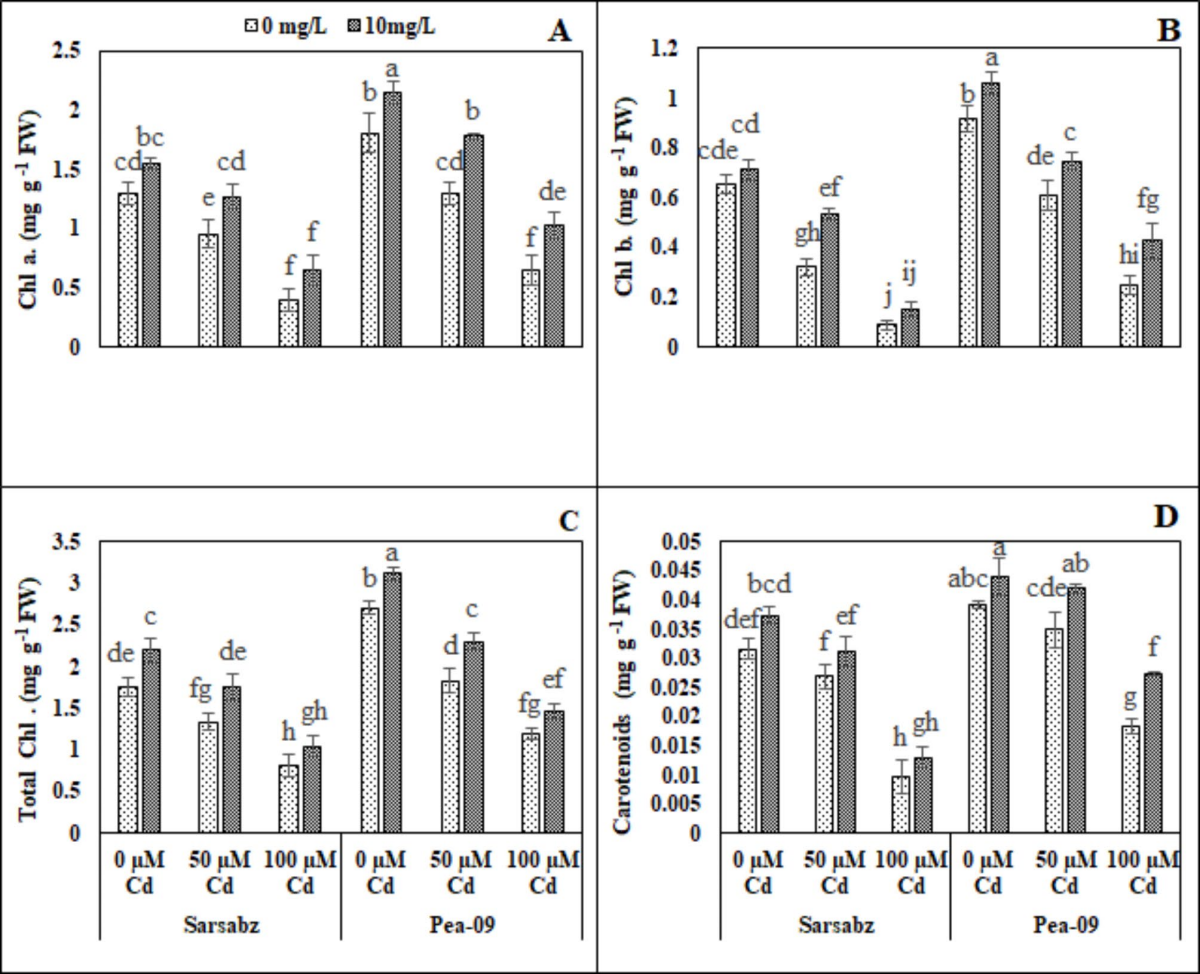
Effect of different levels of Fe − lys (0 and 10 mg L^− 1^) on chlorophyll a (**A**), chlorophyll b (**B**), total chlorophyll (**C**), and carotenoids (**D**) under the toxic concentration of Cd (0, 50 and 100 µM) in the soil in both cultivars of canola (Sarbaz and Pea − 09). All the data represented are the average of four replications (n = 4). Error bars represent the standard deviation (SD) of dour replicates. Different lowercase letters on the error bars indicate a significant difference between the treatments

We also demonstrated various photosynthetic pigments in both cultivars (Sarbaz and Pea − 09) of canola grown under the toxic concentration of Cd (50 and 100 µM) in soil with or without the foliar application of Fe − lys (Fig. 2; Table 1). Results showed that plants which were grown in the toxic amount of Cd in the soil significantly (*P* < 0.05) decreased chlorophyll a, chlorophyll b, total chlorophyll and carotenoid content in both cultivars of canola compared with the plants grown in the control treatment. The concentrations of chlorophyll and carotenoid could be enhanced in both cultivars of canola by the foliar application of Fe − lys which increased these pigments significantly even in the Cd-contaminated soil. Our results showed that the application of Fe − lys increased chlorophyll a, chlorophyll b, total chlorophyll and carotenoid contents by 2.65, 3.64, 6.5 and 4.6%, respectively, in Sarbaz while also increasing by 4.6, 12.6, 15.6 and 5.0%, respectively, in Pea − 09 in the plants which were grown in soil containing the toxic concentration of Cd upon foliar application of Fe − lys compared with plants which were grown without the foliar application of Fe − lys.

### Effect of foliar application of Fe − lys on oxidative stress indicators and antioxidants in both varieties of canola grown under the toxic concentration of cd in the soil

**Fig. 3:**
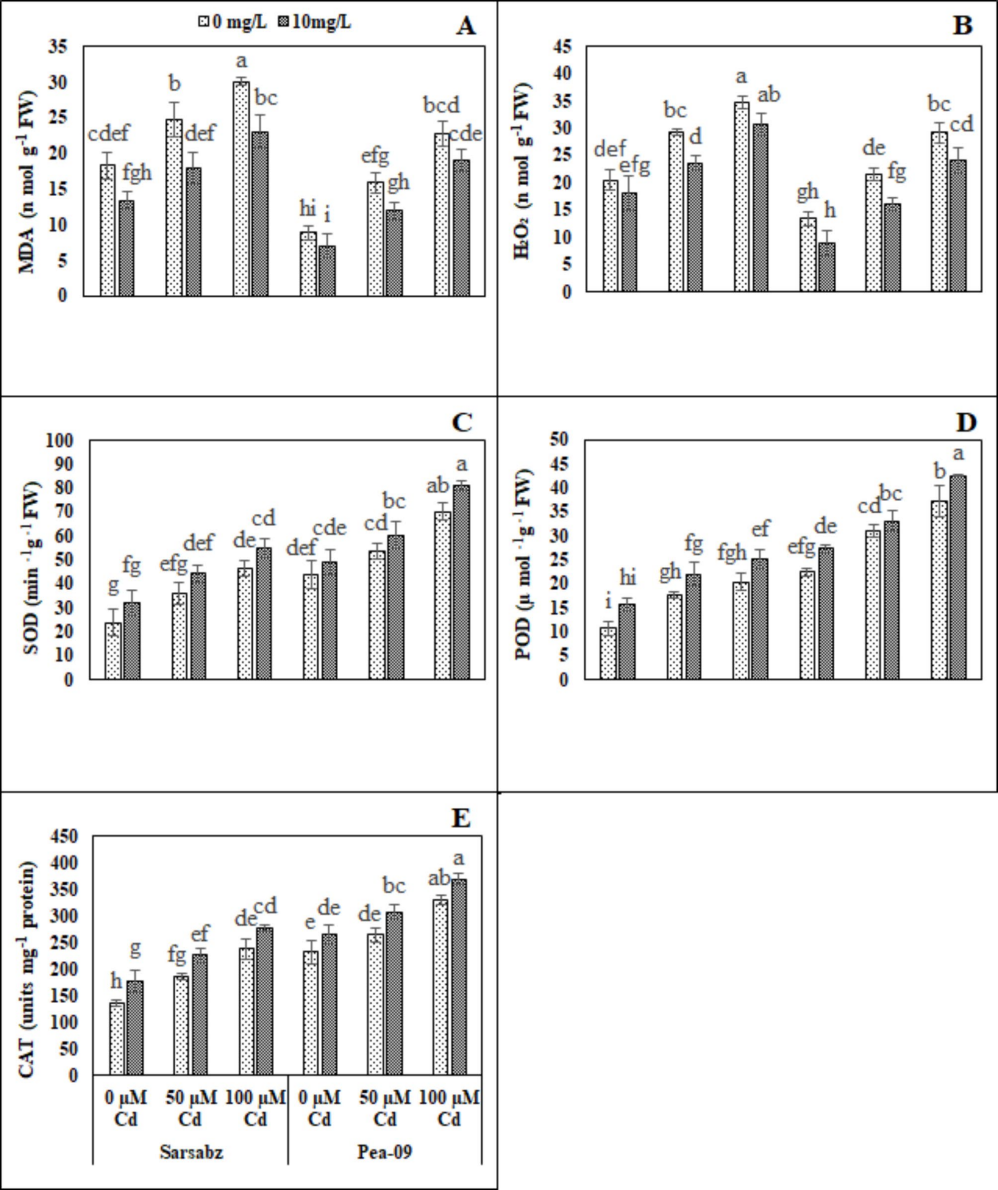
Effect of different levels of Fe − lys (0 and 10 mg L^− 1^) on MDA (**A**), H_2_O_2_ (**B**), SOD (**C**), POD (**D**), CAT (**E**), under the toxic concentration of Cd (0, 50 and 100 µM) in the soil in both cultivars of canola (Sarbaz and Pea − 09). All the data represented are the average of four replications (n = 4). Error bars represent the standard deviation (SD) of dour replicates. Different lowercase letters on the error bars indicate a significant difference between the treatments

We have also measured various oxidative stress biomarkers such as malondialdehyde (MDA) and hydrogen peroxide (H_2_O_2_) contents in canola cultivars under the toxic concentration of the Cd in the soil with or without the foliar application of Fe − lys (Fig. 3; Table 1). Results showed that the toxic concentration of Cd in the soil significantly (*P* < 0.05) increased the contents of MDA and H_2_O_2_ in both cultivars of canola, compared with the plants which were grown in normal soil (Fig. 3; Table 1). Similarly, result showed that the activities of various antioxidants such as superoxide dismutase (SOD), peroxidase (POD) and catalase (CAT) were also increased in the plants which were grown in soil with the toxic concentration of Cd (100 µM), compared with the plants which were grown in the Cd free soil (Fig. 3; Table 1). We also provided evidence that the application of Fe − lys significantly (*P* < 0.05) decreased the contents of MDA and H_2_O_2_ in both cultivars of canola, compared with the plants which were not treated with Fe − lys. In contrast, results additionally illustrated that the application of Fe − lys further increased the activities of various antioxidants (SOD, POD and CAT) in both cultivars of canola, compared with the plants which were not treated with Fe − lys.

### Effect of foliar application of Fe − lys on non-enzymatic compounds and sugar in both varieties of canola grown under the toxic concentration of cd in the soil

**Fig. 4:**
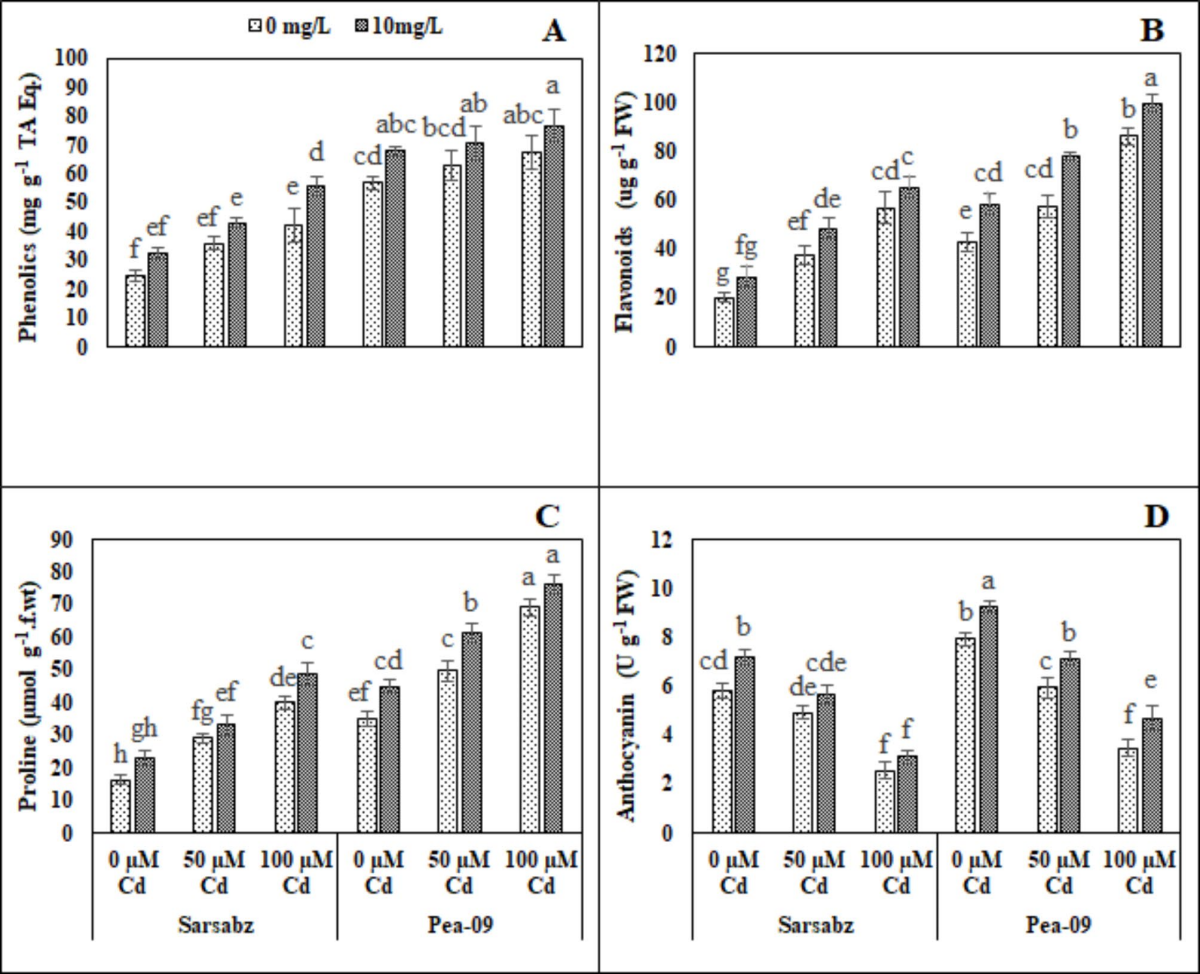
Effect of different levels of Fe − lys (0 and 10 mg L^− 1^) on phenolics (**A**), flavonoids (**B**), proline (**C**) and anthocyanin (**D**) under the toxic concentration of Cd (0, 50 and 100 µM) in the soil in both cultivars of canola (Sarbaz and Pea − 09). All the data represented are the average of four replications (n = 4). Error bars represent the standard deviation (SD) of dour replicates. Different lowercase letters on the error bars indicate a significant difference between the treatments

**Fig. 5:**
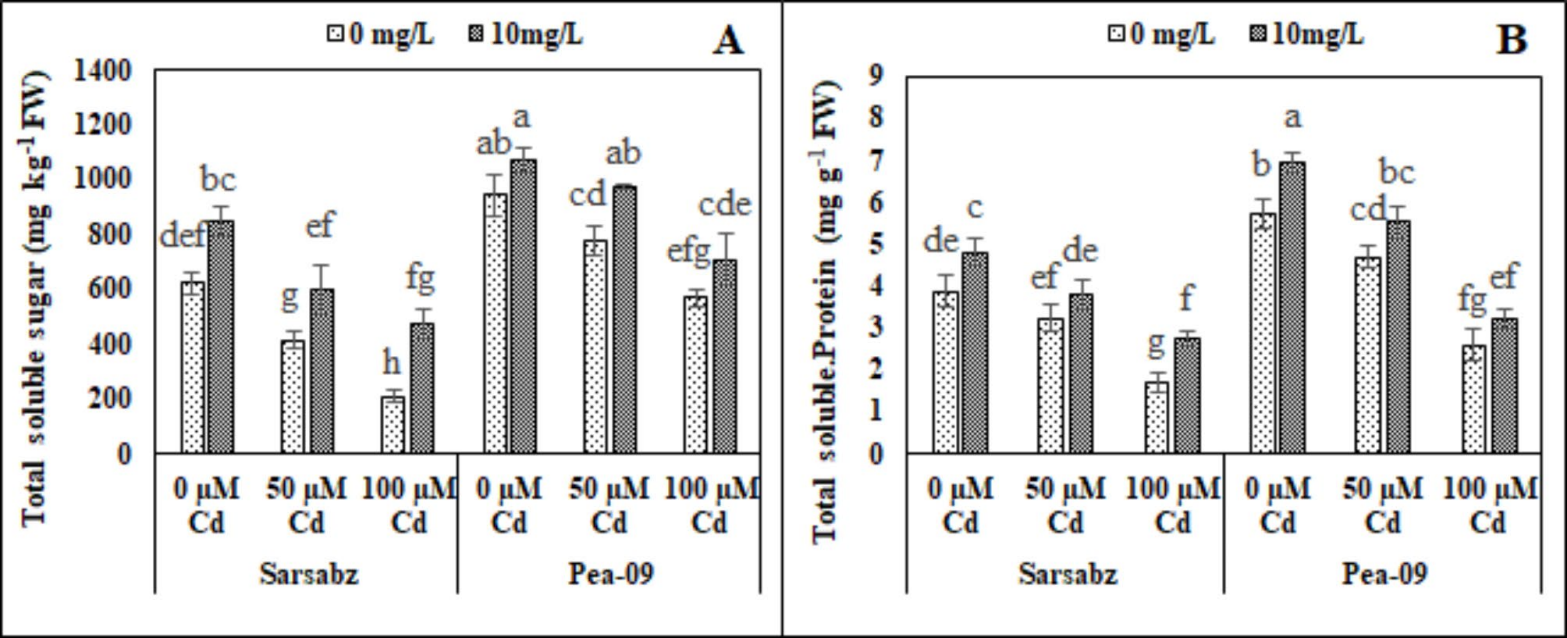
Effect of different levels of Fe − lys (0 and 10 mg L^− 1^) on total soluble sugar (**A**) and total soluble protein (**B**) under the toxic concentration of Cd (0, 50 and 100 µM) in the soil in both cultivars of canola (Sarbaz and Pea − 09). All the data represented are the average of four replications (n = 4). Error bars represent the standard deviation (SD) of dour replicates. Different lowercase letters on the error bars indicate a significant difference between the treatments

In the present study, osmolytes such as phenolics, flavonoids were increased under the Cd stress while anthocyanin and protein and also increased due to the Cd stress (Figs. 4 and 5; Table 1). However, proline contents were increased in both cultivars of canola due to the varying levels of Cd in the soil. Although, our results indicated that the application of Fe − lys increased phenolics, flavonoids, proline, anthocyanin, soluble sugar and soluble protein by 34.79, 23.14, 25.3, 24.2, 18.6 and 20.23% respectively in Sarbaz while increased by 16.55, 26.67, 24.65, 17.6, 20.64 and 34.68% respectively in Pea − 09.

## Discussion

Heavy metals including Cd are considered as one of the environmental toxic factors that directly affect the plant growth and yield. Therefore, the response of plant growth and biomass has become the major morphological phenomenon under Cd stressed environment [16, 53, 54]. In the present study, we elucidated that growth and biomass of canola were significantly (*P* < 0.05) decrease when subjected to Cd-polluted regimes (Fig. 1; Table 1). Decreased plant growth and biomass under excess Cd levels in plants has already been reported phenomenon [55–57] which depends upon number of environmental factors including plant species, treatments, growth medium and soil Cd contents [58, 59]. Reduction in growth and biomass accumulation could also be attributed to alterations in the ultrastructure of various plant components under toxic levels of Cd in sand that have direct impacts on plant growth and yield [59–61]. Present study revealed that photosynthetic pigments (Fig. 2; Table 1) of canola varieties (Fig. 2; Table 1) were significantly (*P* < 0.05) affected under increasing levels of Cd (0, 50 and 100 µM) treatments. Excessive Cd concentrations affected the chlorophyll pigments due to two important factors; (i) stomatal factors and (ii) non-stomatal factors [62, 63]. Excess Cd levels are generally known to initiate oxidative stress in plants by production of extra reactive oxygen species (ROS) [16, 64] and antioxidative enzymes play a protective role in reducing the metal toxicity by scavenging ROS [65, 66]. Previously, excess amount of Cd increased ROS production in cells/tissues, which were then scavenged by the activities of antioxidant compounds [13, 67] and osmolytes [68, 69]. The increase in the activities of antioxidant enzymes was concomitant with the generation of extra ROS [70]. It was also reported that increased in the activities of various antioxidant enzymes under environmental stress condition is also due to the reduction in glutathione contents [71, 72]. Plant cells provide a space under conditions of oxidative stress where signal transduction can be carried out by the formation of proline, which is a vital response by plants against Cd toxicity [59, 73].

Various plant growth hormones have been widely used recently to mitigate oxidative stress and prompt plant growth and composition in different plant species when grown in metal-polluted soil [26, 74]. Although, the usage of micronutrients with the amino acid complex is an incredibly new idea and showed beneficial results such as improved plant growth and composition and restrict the plant for uptake/accumulate toxic content in their body parts [75, 76]. There is also some literature on various plant species, which, when grown on metal contaminated soil, alleviates metal toxicity and improves plant growth and biomass [68, 77]. Amino acids are simple organic compounds, which constitutes proteins and it has been reported that amino acids chelated with micronutrients may alleviate abiotic stress in plants [78, 79]. Fe is the essential micronutrients for plants, however, and plants need to take them externally to sustain their body’s normal growth and development [21, 22, 80]. Recently, this technique has become interestingly popular in alleviating heavy metal stress in different plant species and used for enhancing plant growth and biomass [19, 21, 81]. The plant can also uptake amino acids from the soil, which also plays a crucial role in the physiological mechanisms (such as photosynthesis) of the plants [22, 82]. It is well-known that foliar application of Fe − lys decreased oxidative stress in the plants, when grown with or without abiotic stress conditions [41, 75]. This is because amino acids has the efficiency for scavenging ROS production and decreasing oxidative stress in a number of plant species [83, 84]. Although Fe chelated with lys increased the activities of various antioxidant enzymes, plays a protective role by decreasing the contents of Cd in various parts of the plants.

## Conclusion

On the basis of these findings, it can be concluded that the negative impact of Cd toxicity can be overcome by the application of Fe − lys. Moreover, our results depicted that Cd toxicity induced severe metal toxicity in canola cultivars by increased generation of ROS in the form of oxidative stress, which ultimately decreased plant growth and yield and photosynthetic efficiency. Hence, Cd toxicity was eliminated by the external application of Fe − lys, which also degenerated ROS, and increased the activities of antioxidants. Therefore, long-term field studies should be executed to draw parallels among plants and crops root exudations, metal stress, nutrient mobility patterns, and plant growth in order to gain further insights into underlying mechanisms.

## Data Availability

All data generated or analysed during this study are included in this published article.
